# Empyema necessitans in a pediatric patient: A case study

**DOI:** 10.1016/j.ijscr.2023.108932

**Published:** 2023-10-14

**Authors:** Yusuf Shieba, Alaa Ramadan

**Affiliations:** a Faculty of Medicine, South Valley University, Qena, Egypt; b Cardiothoracic Surgery Department, Qena, Egypt

**Keywords:** Empyema necessitans, *Escherichia coli*, Pediatric, Case report

## Abstract

**Introduction and importance:**

Empyema necessitans is a rare and severe complication of pleural effusion characterized by the extension of purulent material from the pleural cavity into the surrounding soft tissues, resulting in the formation of a subcutaneous abscess.

**Case presentation:**

A one-year-old boy presented with symptoms that were in line with empyema necessitans, and *Escherichia coli* was shown to be the causative organism. A successful outcome required early detection, rapid diagnosis, and proper management, which included targeted antibiotic medication and drainage of the pleural collection. When a young patient exhibits a growing chest wall swelling, empyema necessitans should be considered in the differential diagnosis.

**Clinical discussion:**

The best care for individuals with empyema necessitans requires a multidisciplinary approach comprising pediatricians, thoracic surgeons, infectious disease experts, and interventional radiologists. *Escherichia coli* infection requires a comprehensive approach involving antibiotic therapy and surgical intervention if necessary.

**Conclusion:**

Empyema necessitans in pediatric patients, caused by *Escherichia coli*, is an infrequent disease that requires more investigation to enhance our understanding of the associated risk factors, optimal treatment modalities, and potential long-term consequences.

## Highlights

• When a young patient exhibits a growing chest wall swelling, empyema necessitans should be considered in the differential diagnosis.
• Empyema necessitans in pediatric patients, caused by *Escherichia coli*, is an infrequent disease that requires more investigation to enhance our understanding of the associated risk factors.
• The best care for individuals with empyema necessitans requires a multidisciplinary approach comprising pediatricians, thoracic surgeons, infectious disease experts, and interventional radiologists.

## 1 Introduction

Empyema necessitans is a rare and potentially life-threatening complication of empyema, characterized by the extension of purulent material from the pleural cavity into the surrounding soft tissues, resulting in the formation of a subcutaneous abscess. It is most commonly observed in patients with chronic or inadequately treated empyema, but it can also occur as a primary infection [1].

Usually, a subpleural abscess ruptures through the chest wall, allowing the infected material to track through the intercostal gaps and dissect along the fascial planes and resulting in empyema necessitans. The pathogenic organisms, such as *Streptococcus pneumoniae*, *Staphylococcus aureus*, and anaerobic bacteria, are typically comparable to those found in empyema. Mycobacterial or fungal infections, however, may occasionally be the cause of this illness [2,3].

Depending on the length and intensity of the infection, empyema necessitans can manifest clinically in a variety of ways. Patients frequently present with a subcutaneous lump or swelling, usually in the upper back or chest wall. Typically, hard and sensitive, the swelling may also be accompanied by erythema and warmth. The abscess may fluctuate as the infection worsens and eventually drain on its own through a fistulous channel, releasing purulent material [4,5].

The treatment of empyema necessitans is multidisciplinary, with the goals of controlling the infection, alleviating symptoms, and preventing consequences. The cornerstone of treatment is a combination of proper antibiotic medication and abscess drainage. Empiric antibiotic therapy is commenced based on the presumed causal organisms and is adjusted depending on culture and sensitivity data. Depending on the clinical circumstances and the patient's health, drainage is often accomplished by either percutaneous catheter implantation, surgical drainage, or a mix of both [6,7].

Surgical intervention may be necessary in cases where percutaneous drainage is ineffective or in the presence of complications such as bronchopleural fistula, lung necrosis, or organizing phase of empyema. Surgical procedures may range from thoracotomy with decortication and complete evacuation of the abscess to minimally invasive techniques like video-assisted thoracoscopic surgery (VATS) [8].

The choice of surgical approach depends on the individual patient's characteristics, the extent of the infection, the stage of empyema and the experience of the surgical team. The prognosis of empyema necessitans is determined by the promptness of diagnosis, the efficacy of treatment, and the patient's overall condition. The majority of patients can recover completely if they receive prompt and adequate treatment. However, a delayed diagnosis or insufficient treatment might result in consequences such as chronic sinus formation, septicemia, or even death [3,6]. This case report has been reported in line with the SCARE criteria [9].

## 2 Case presentation

A one-year-old male patient presented at the cardiothoracic surgery department of South Valley University Hospital with right-sided chest swelling for 4 days duration associated with non-radiating pain. The chest swelling was gradual in onset, with a progressive course. His mother presented that he had a history of productive cough for two weeks duration. Three weeks prior, he had been hospitalized with a urinary tract infection. There were no systemic symptoms, rash, or chills. There was no history of chest trauma or previous surgery.

**Fig. 1:**
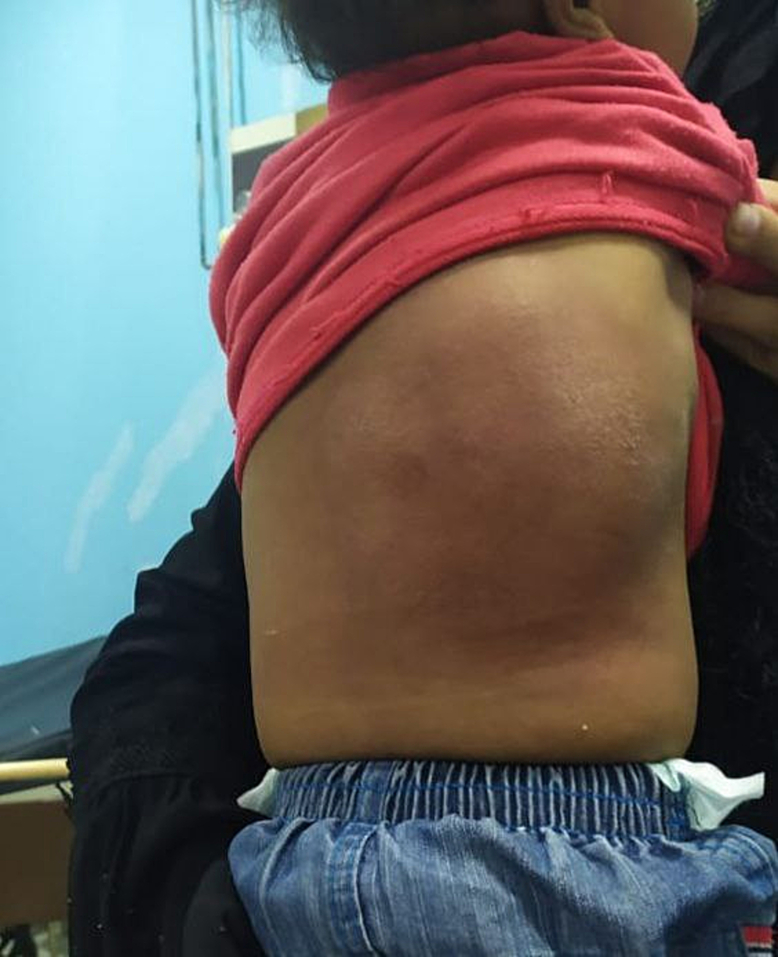
Bulging mass on the right region of the chest wall.

On examination, the patient appeared ill, with increased work of breathing. His temperature was 38.8 °C, heart rate was 120 beats per minute, and respiratory rate was 43 breaths per minute. Oxygen saturation was 94 % on room air. Inspection of the chest revealed a prominent, erythematous, firm, and tender swelling in the right back of the chest, measuring approximately 10 cm in diameter. The overlying skin was warm, with evidence of fluctuation without spontaneous discharge Fig. 1.

**Fig. 2:**
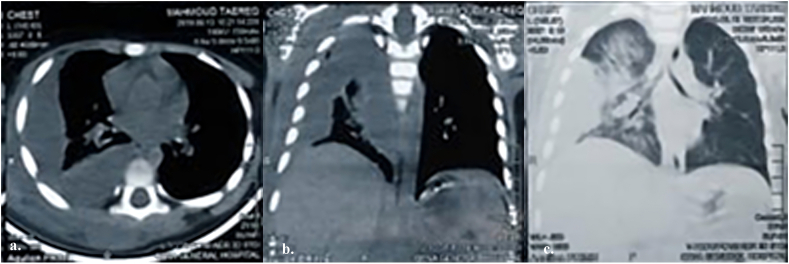
Ct Chest without contrast showing Rt- sided encysted empyema and thick pleura; a) cross-sectional View: An empyema refers to the collection of pus within a pre-existing space; b) Coronal View - Mediastinal Window: The empyema and thickened pleura on the right side can be visualized in relation to the mediastinal structures; c) Coronal View - Pulmonary Window: The empyema and thickened pleura on the right side can be seen in the context of the lung tissue.

A chest X-ray revealed a significant amount of right-sided pleural effusion and a wholly opaque right lung. The subsequent chest CT scan confirmed the presence of Rt lower lobe consolidation and loculated pleural effusion extending along the lateral and posterior chest wall Fig. 2.

Leukocytosis (white blood cell count of 18,000 cells/mm^3^) was discovered by laboratory testing. To identify the causative organism, a sample of the pleural fluid was collected for microbiological analysis. Gram staining showed numerous polymorphonuclear leukocytes and gram-negative bacilli. Culture of the pleural fluid grew *Escherichia coli*, confirming the diagnosis of empyema necessitans due to an *Escherichia coli* infection. Based on the isolated strain's susceptibility patterns, we initiated the empiric antibiotic therapy with intravenous cefotaxime 200 mg/kg/day and intravenous vancomycin 60 mg/kg/day to cover the most likely pathogens, such as *Streptococcus pneumoniae* and *Staphylococcus aureus*. And Once culture and sensitivity results become available, the patient was immediately started on the appropriate intravenous antibiotics. The third-generation cephalosporin was a component of the antibiotic regimen.

**Fig. 3:**
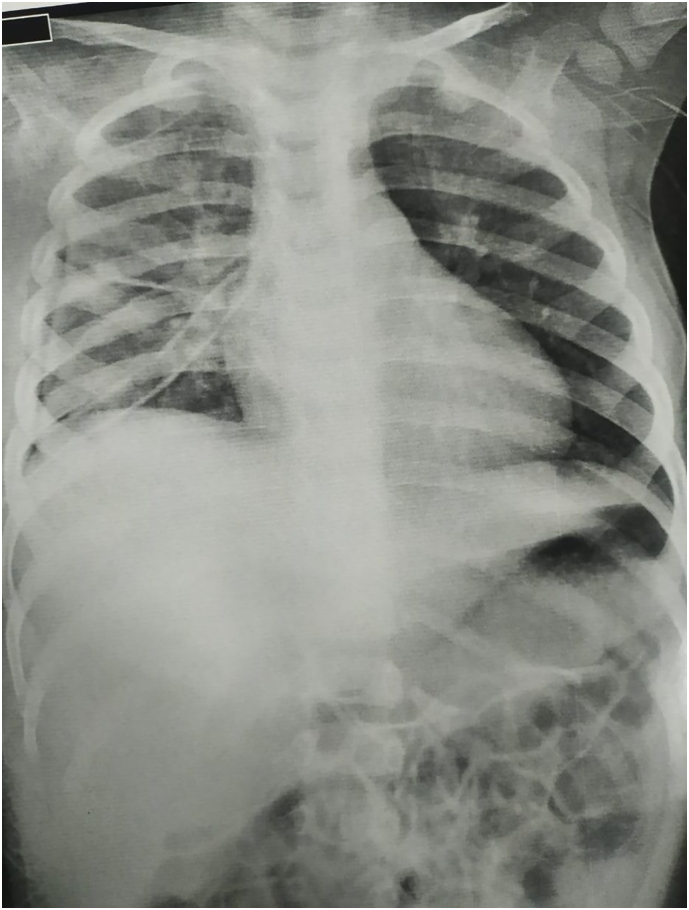
Follow up chest x-ray showing chest tube in place.

Under general anesthesia, the patient underwent surgical drainage, the abscess loculation was broken down effectively in all directions, and yellowish-white pus was drained; thereafter, the abscess cavity was thoroughly irrigated with sterile saline solution. Moreover, the abscess cavity was packed with sterile gauze. Lastly, a thoracostomy tube was inserted to drain the pleural cavity. Then patient was admitted to ICU for close monitoring, respiratory support, and postoperative care. The child's clinical course was constantly observed, and laboratory values, vital signs, and respiratory status were evaluated on a regular basis. Throughout the hospital stay, the patient gradually displayed clinical improvement. The swelling on the right side of his chest gradually went down in size as his fever reduced, respiratory symptoms was improved. Follow-up imaging revealed that the pleural effusion had resolved, and the lung regain its expansion. After 14 days, the patient successfully finished the intravenous antibiotic regimen and was discharged in stable condition. At the 1-month follow-up, the child remained asymptomatic with no recurrence of infection Fig. 3.

## 3 Discussion

Empyema necessitans is a rare medical condition characterized by the development of a purulent infection in the soft tissues due to a connection between an empyema and the chest wall. This typically occurs in cases of persistent pneumonia [10]. Pneumonia caused by *Escherichia coli* (*E. coli*) is an uncommon condition. Gram-positive bacteria, such as *Streptococcus pneumoniae* and *Staphylococcus aureus*, are responsible for the majority of cases of community-acquired pneumonia; however, gram-negative bacteria are becoming increasingly common [11]. Frequently, pleural infection is secondary to pulmonary infection. 15–44 % of admitted patients with pneumonia develop pleural effusion, and 40 % of these patients are compounded by parapneumonic effusion or abscess [12]. The presented patient had a history of urinary tract infection three weeks prior to the onset of respiratory symptoms. In addition, the chest CT scan demonstrated lower lobe pneumonia. In the case we mentioned, *E. coli* was identified as the causative organism. Additionally, there is an association with other Gram-negative organisms. According to Nwagboso et al. study, the most common Gram-negative organism isolated from the pleural aspirate samples of patients diagnosed with empyema in Nigeria was *Klebsiella pneumoniae*. Therefore, targeted empirical antibiotic therapy based on local microbiology and antibiotic resistance patterns is essential for the treatment of empyema of different organisms [13].

*Escherichia coli* is a gram-negative bacterium commonly found in the gastrointestinal tract or urinary tract and is known for its pathogenic potential. Patients who have empyema necessitans caused by an infection with *Escherichia coli* frequently exhibit symptoms like fever, coughing, chest pain, and respiratory distress [14].

Individuals at risk of developing *E. coli* pneumonia encompass those with concurrent medical conditions, including immunosuppression (31.7 %), diabetes mellitus (18.5 %), persistent alcohol consumption (23 %), chronic respiratory ailments (13.6 %), and chronic heart failure (17.7 %). Additionally, it was observed that 57 % of the *E. coli* strains found in pneumonia patients originated from urinary sources [15].

Upon physical examination, the affected area frequently has a visible and palpable subcutaneous swelling that may be warm, erythematous, and painful. There may also be systemic indicators of illness, such as tachycardia and accelerated breathing. Children may exhibit nonspecific signs such irritability, poor eating, and failure to thrive. Clinical evaluation, imaging tests, and laboratory testing are all used in the diagnostic evaluation of empyema necessitans caused by *Escherichia coli* infection. A chest X-ray is typically the first imaging technique used, and it may show that the affected hemithorax has become opaque and that there is a loculated pleural fluid collection present. A computed tomography (CT) scan can reveal more precise details on the severity of the illness, including whether or not soft tissues are affected [14,16]. To obtain pleural fluid samples for use in confirming the presence of infection and identifying the causal organism through microbiological cultures, diagnostic thoracentesis is essential. A thorough strategy comprising antibiotic therapy and, if necessary, surgical intervention is required for the management of empyema necessitans brought on by an infection with *Escherichia coli*. Following a fast start to empirical antibiotic therapy, *Escherichia coli* and other possible infections are targeted based on the patient's age and the local resistance trends. Antimicrobial susceptibility testing helps determine which antibiotics to use. For both diagnostic and therapeutic reasons, it is imperative to drain the contaminated pleural fluid [8,15].

The purulent material can be effectively evacuated, and the empyema can be resolved by inserting a chest tube or percutaneous catheter under imaging guidance. In situations where loculated collections exist or when conservative therapy has failed, surgery such as VATS or thoracotomy may be required. *Escherichia coli* infection-induced empyema necessitans can produce a number of consequences, such as sepsis, respiratory failure, and the development of bronchopleural fistulas [17].

These complications can arise from either a delayed diagnosis or insufficient therapy. For a full recovery and to avoid complications, prompt and proper management, together with careful observation of the patient's clinical development, imaging tests, and follow-up visits, are crucial. The prognosis for patients with empyema necessitans caused by *Escherichia coli* infection can be favourable with prompt treatment, while individual characteristics and the existence of any underlying disorders may have an impact on the final result [3,18].

## 4 Conclusion

*Escherichia coli* infection-induced empyema necessitans is a rare and difficult disorder, especially in pediatric populations. For patient outcomes to be maximized, early detection, precise diagnosis, and timely application of the proper treatment are essential. To offer comprehensive care, a multidisciplinary approach including healthcare specialists from many specialties is required. To enhance the treatment and prognosis of empyema necessitans brought on by an infection with *Escherichia coli*, more study and improvements in diagnostic procedures, therapeutic interventions, and preventive measures are required.

## Ethical approval

The study was exempt from ethnical approval in my institution as the data used in this report can be accessed with the explicit consent obtained from the patient involved.

## Funding

No funds.

## Author contribution

Y.S. study concept or design, data collection, writing the paper.

A.R. writing the paper.

## Guarantor

Yusuf Shieba.

## Research registration number

Not applicable.

## Conflict of interest statement

No conflict.
